# Long-Term Outcomes after Phthalate Exposure: Food Intake, Weight Gain, Fat Storage, and Fertility in Mice

**DOI:** 10.1289/ehp.120-a320a

**Published:** 2012-08-01

**Authors:** Wendee Holtcamp

**Affiliations:** Wendee Holtcamp, based in Houston, TX, has written for *Nature*, *Scientific American*, *National Wildlife*, and other magazines.

Exposure to endocrine-disrupting chemicals (EDCs), particularly *in utero*, is suspected to contribute to obesity, diabetes, hypertension, and reproductive abnormalities. Di(2-ethylhexyl) phthalate (DEHP), a plasticizer found in cosmetics, fragrances, food packaging, and polyvinyl chloride, is one such EDC. Human studies have found associations between urinary metabolites of DEHP and other phthalates and increased body mass in humans, and maternal exposure to DEHP has been associated with impaired gonadal development and fertility in baby boys. However, much less is known about potential effects of DEHP on female health. In a two-part investigation, researchers documented weight and fertility changes in female mice exposed to DEHP, and then documented how exposure *in utero* and during lactation affected their offspring [*EHP* 120(8):1123–1129; Schmidt et al.].

In the first study, adult female mice were given diets formulated to deliver one of three levels of DEHP (0.05, 5, or 500 mg/kg body weight) for 8 weeks. The lowest level was comparable to the tolerable daily intake for humans issued by the World Health Organization in 2003. Although outwardly healthy, dams fed all three levels of DEHP had significantly increased food intake, body weight, and visceral fat compared with controls. All treatment groups also showed increased gene expression of the hormone leptin (consistent with the animals’ increased visceral fat) and decreased expression of adiponectin (which may suggest potential effects on insulin sensitivity).

As in previous studies, investigators found that at the highest level of DEHP exposure, expression of peroxisome proliferator-activated receptors alpha and gamma (PPARα and PPARγ)—which are mediators of adipogenesis and fat metabolism—was increased in the liver. However, unlike previous studies, they found a decrease in PPARα expression in visceral fat at the highest dose. The authors note that a pair-feeding design in which exposed and control animals receive the same amount of calories should be used to determine whether observed effects were a consequence of increased food intake or of metabolic effects of DEHP independent of caloric intake.

In the second study, the investigators found that DEHP-exposed pups of both sexes had higher body weight at weaning than nonexposed pups. Higher body weight persisted 9 weeks after exposure ceased, and fat storage was significantly higher in female adult pups in a dose-dependent manner. These findings were surprising because DEHP is rapidly metabolized and excreted, yet the results suggest a lingering effect of *in utero* exposure to DEHP on body weight and fat tissue formation.

At the highest DEHP exposure, a dose unlikely to be found in the environment, all dams experienced 100% spontaneous abortion. Surviving, lesser-exposed offspring were placed on a standard diet at weaning, and female offspring were mated to unexposed males. Although the total number of embryos was not reduced in pregnant females exposed to DEHP *in utero*, the investigators did find that 28% of the dams’ blastocysts were not viable in the low-dose group and 29% were not viable in the middle-dose group, compared with just 8% in controls. However, the difference was not statistically significant.

